# The Use of Silver Iodide in Urethritis and Cystitis

**Published:** 1912-01

**Authors:** James A. Gardner

**Affiliations:** Buffalo. N. Y.


### The Use of Silver Iodide in Urethritis and Cystitis

By JAMES A. GARDNER, M.D.
Buffalo, N. Y.

SILVER iodide is a preparation for use as an injection for various inflammatory conditions of the urethra and bladder. It was first brought to my attention by an article by Dr. G. Paul Laroque, (1) who quotes from the report of Siter & Uhle. (2). The preparation as used at that time was a ten per cent. suspension of iodide of silver in mucilage of quince seed.

During 1907, I used the preparation in a number of cases but because of the difficulty of preparing it, I stopped using it. It was again brought to my attention by the experimental department of Parke, Davis & Co., which had devised an ingenious method of furnishing a silver iodide so that physicians may prepare their own dilutions extemporaneously at the time required and which preparations can be mixed in an aqueous solution. This preparation is a finely palpable powder which is in suspension rather than in solution. With it treatment is carried out with very little difficulty. It has all the known advantages of the ordinary silver salts with none of their annoying disadvantages. With the exception of argyrol all of the silver salts with which I am familiar and which are claimed to be non-irritating, cause more or less complaint by patients when they are used in sufficient strength to bring about results. With argyrol, as we all appreciate, the great disadvantage is the staining of one's hands and the patient's linen.

It is claimed that silver iodide does not coagulate albumen and it apparently is an efficient germicide and is non-irritating unless used in strength of 1-10 or stronger. The preparation which is furnished the physician is a concentrated syrupy solution and is first prepared by the admixture of potassium iodide and silver nitrate with a small amount of glycerine and water. This forms a clear solution and when the physician wishes to use the preparation he measures the necessary quantity of water and then by means of a dropper or pipette he adds to the water a very small quantity of the concentrated silver iodide solution. When this concentrated solution is diluted with water the silver iodide is precipitated in suspension for some time. The finely divided silver iodide in suspension is ready for application by merely shaking up the aqueous solution at the time of injection.

On review of over one hundred cases treated with this preparation: At first according to the instructions given me by the manufacturer I began using solutions about 1-4000 to 1-2000. This preparation was most bland and non-irritating. I then from time to time increased the strength and found that I obtained better and quicker results. I believe that the preparation relieves the inflammatory condition in two ways, first, mechanically by covering the mucus membrane with this powder which settles down after a short time; secondly, by its germicidal action. My routine method of treatment in acute cases is as follows: the discharge is carefully stained to make positive diagnosis for gonococci. At first, I use an ounce syringe with a solution 1-60. I have never seen any complication arise from placing this medication in the deep urethra at the outset of the disease; on the contrary I believe that if we are so fortunate as to be able to cover the mucus membrane before it is attacked by the bacilli, we have made a distinct gain. If the urethra is very much inflamed I precede the silver iodide with a two per cent. solution of cocaine to allay the sensitiveness. This treatment is repeated once a day for three or four days when, as a rule, the patient will make no complaint from the use of the silver iodide alone. At that time he is given a four ounce bottle with two c.c. of silver iodide equalling 1-60; also an ounce syringe with a soft rubber tip with instructions always first to urinate and then slowly to inject, holding the solution for five minutes. This leaves about two-thirds of the solution in the posterior urethra and in the bladder.

That which he has left in the urethra remains there after three or four urinations and that in the bladder has a chance to well coat the posterior urethra thoroughly before the next urination so that we have found silver iodide in the urine forty-eight hours after the application. The patient at this time should be instructed to drink large quantities of water, it often helping him to follow out these instructions if lithium carbonate tablets are prescribed in large glass of water every two hours. If his urination becomes painful that old prescription of

| | |
|---|---|
| Tr. Hyoscyami | oz. 2 |
| Liq. Potassae | drachm. 2 |
| Syrup. Cinnamomi | q. s. ad oz. 4 |

teaspoonful in water every two hours is substituted for the lithia tablet. The patient for a few days still reports once a day when he is given the injection of 1-60 to 1-30 and uses the solution of 1-60 two or three times during the twenty-four hours at home. The average acute case subsides in two or three weeks. When the discharge from the anterior urethra clears up and the prostate and seminal vesicles have been proved not to be infected, an instillation of 10 to 15 minims of a strength of from 1-20 to 1-10 in the Keyes-Ultzman syringe is used. It is understood that any complicating prostatitis, vesiculitis or folliculitis or other complication must be eradicated before the urethritis will clear up.

In using the instillation with preparations of about 1-20 to 1-10 the patient will often complain as they would in using nitrate of silver ½ per cent. strength but after two or three minutes the pain as suddenly subsides as it began, a marked contrast to the use of nitrate of silver. When microscopic examination of the shreds in the urine and the prostatic secretion fails to show Neiser bacilli and there remains only the catarrhal remnants, astringents such as nitrate of silver 1-7500, zinc sulphate or lead acetate may be used as an irrigation. Silver nitrate is probably the most effective drug we have for this purpose.

After finding this product very effective in gonorrhoeal urethritis I also found that it was effective in allaying inflammatory conditions of the non-specific urethritis. I think in these latter cases that it was more from the mechanic effect of covering the mucus membrane than from any germicidal action. In briefly outlining the treatment by the use of silver iodide I have not mentioned the use of balsams or various other internal remedies nor have I mentioned the complications which often arise, strictures, peri-urethral abscesses, chronic folliculitis, chronic prostatitis and vesiculitis, but these conditions of course need to be treated apart from a surface application with drugs. In the treatment of urethritis I think we are all agreed that the more soothing and bland the preparation can be and still be effective, the more we gain. I believe this new preparation which is remarkable clean, is effective first, mechanically, by covering the irritable mucus membrane, and, second, by its germicidal action and last but not least, is most bland and non-irritating.

403 FRANKLIN STREET.

---

1. Therapeutic Gaz., Nov. 15, 1906.
2. University of Penna. Medical Bulletin, May, 1905.

